# Supplementary material for: Exogenous dsRNA triggers sequence-specific RNAi and fungal stress responses to control Magnaporthe oryzae in Brachypodium distachyon
Source: Commun Biol. 2025 Jan 25;8:121. doi: 10.1038/s42003-025-07554-6 (PMC11762700; doi:10.1038/s42003-025-07554-6)
Supplement: Supplementary file 2 — Supplementary Information [file 42003_2025_7554_MOESM2_ESM.pdf]

Exogenous dsRNA triggers sequence-specific RNAi and fungal stress responses to control *Magnaporthe oryzae* in *Brachypodium distachyon*

Ying Zheng[1], Benjamin Moorlach[2], Desiree Jakobs-Schönwandt[2], Anant Patel[2], Chiara Pastacaldi[1], Stefan Jacob[3], Ana Sede[4], Manfred Heinlein[4], Minna M. Poranen[5], Karl-Heinz Kogel[4*], Maria Ladera Carmona[1*]

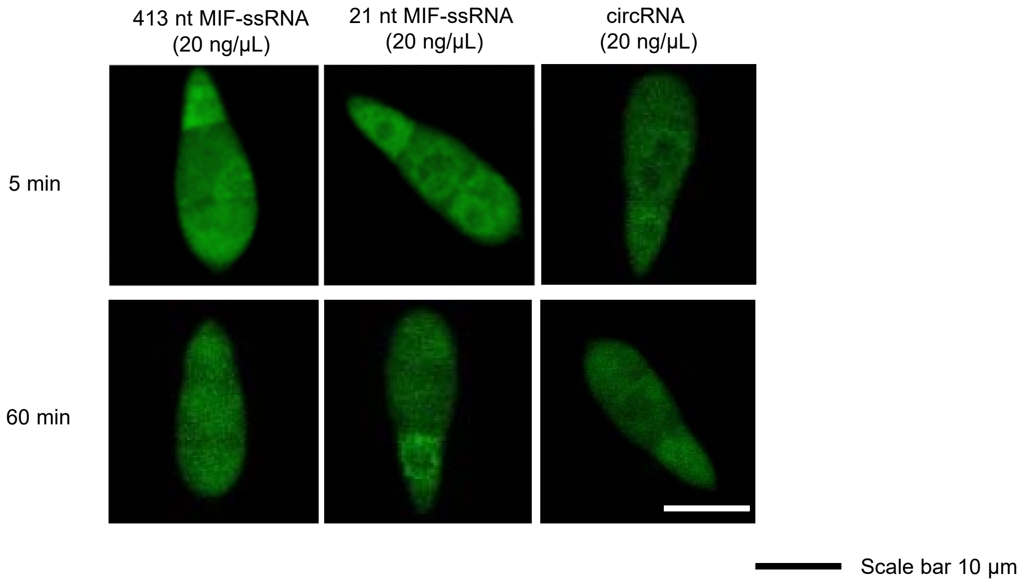

Figure S1. Imaging nuclear transfer of chimeric GFP-MoHog1p in conidia of *Magnaporthe oryzae* in response to different stressors. Conidia (2000/100 µL) in Tween water were incubated with 20 ng/µL of the respective RNA. Pictures were taken with the AF488 laser at 5 and 60 min after treatment. Scale bar equals 10 µm. AF488 [λexcitation (nm): 492; λemission (nm): 561]. ss- single-stranded RNA; circ- circular RNA.

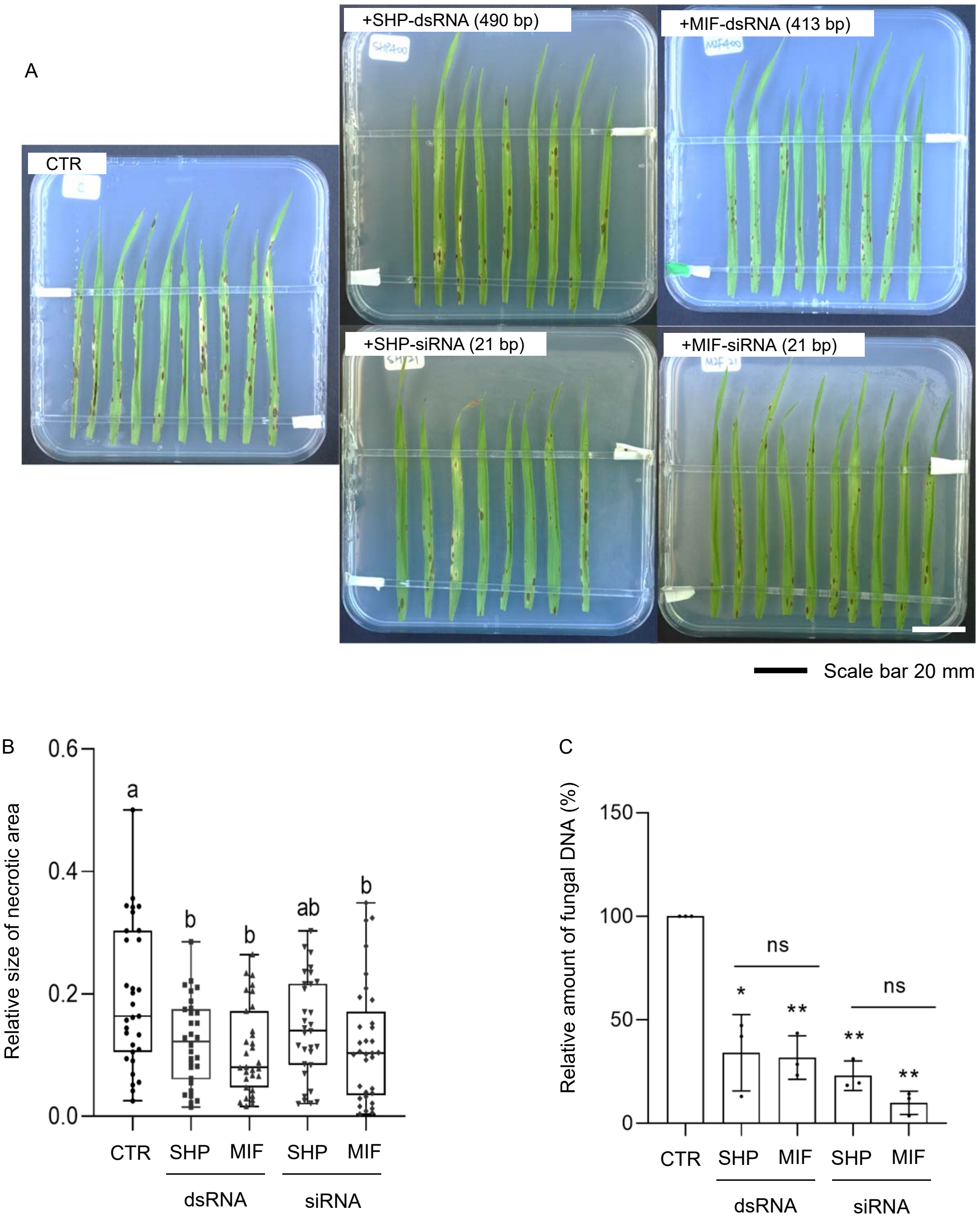

Figure S2. RNA-spray-mediated protection of *Bd* plants against *Mo* infection. (A) Infection symptoms on *Bd* leaves inoculated with a mixture of *Mo* conidia and dsRNA or siRNA. Intact three-week-old *Bd* plants were sprayed with a solution containing conidia (65 × 10^3 conidia mL^{-1}) and 10 ng/µL dsRNA/siRNA as indicated in the figure. Control plants were sprayed only with conidia solution (CTR). For disease symptom evaluation, second youngest leaves were placed on 1% agar plates at 5 dpi and unfolded with the help of a plastic stick for better image acquisition. A representative image is shown. Scale bar = 20 mm. (B) Relative size of the necrotic area compared to the whole leaf area calculated with ImageJ. Box plots show the mean with standard deviation of three independent repetitions. Statistical significance was assessed with one-way ANOVA ($p \leq 0.05$) and significance letters were assigned according to Tukey's multiple comparisons test. (C) The levels of the fungal housekeeping gene *MoGPD* were quantified by qPCR and normalized using *Bd* housekeeping gene *BdUbi10*. The percentage of relative amount of fungi in comparison to the control from three independent repetitions was combined, represented as average with standard deviation. Statistical significance was assessed with two-tailed *t*-test ($p \geq 0.05$) and one sample *t*-test to the control group ($p \leq 0.05$). (*: p=0.0253; ** MIF-dsRNA: p=0.0078; ** SHP-siRNA: p=0.0028; ** MIF-siRNA: p=0.0013).

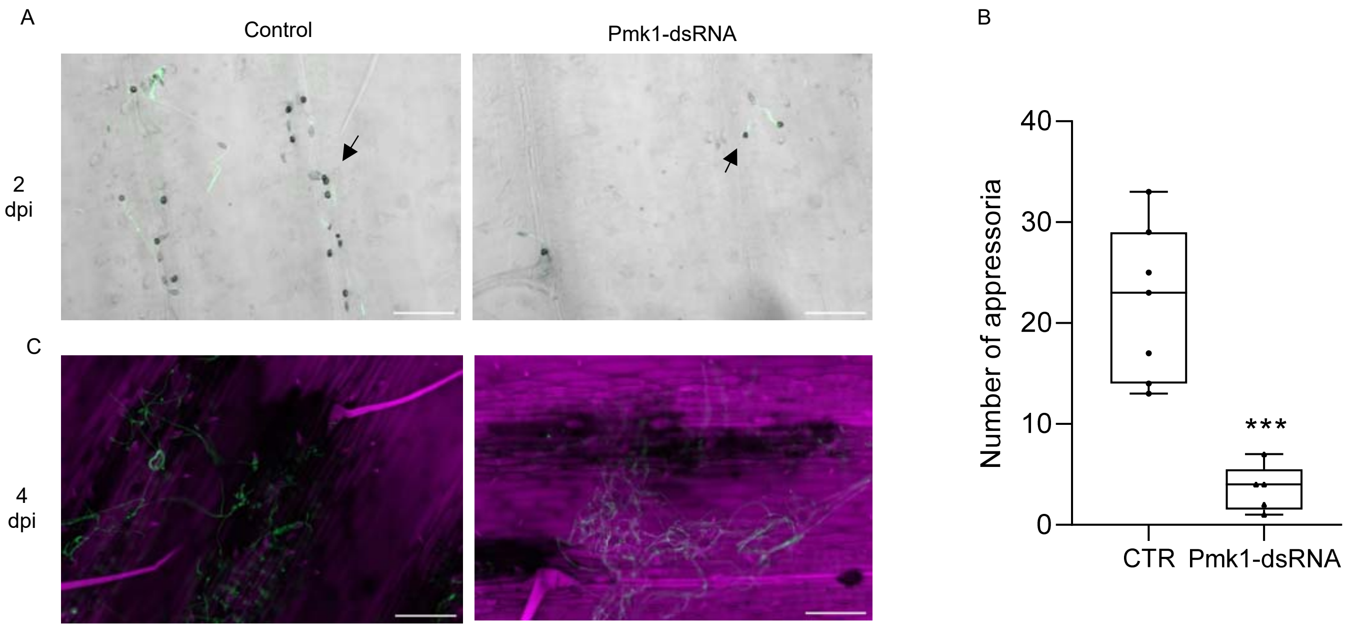

Figure S3. Conidia treated with Pmk1-dsRNA are impaired in leaf penetration. Detached *Bd* leaves were treated with a solution containing conidia in tween water (200 conidia in a 20 µL drop) or conidia with Pmk1-dsRNA (10 ng/µL). (A) Merge pictures of AF488-WGA staining [λexcitation (nm): 485 +/- 20; λemission (nm): 515] of *Mo* fungal structures at 2 dpt. Arrows indicate an appressorium. One representative picture of each treatment is shown. (B) Appressoria quantification in control and Pmk1-dsRNA treated samples. Box plots represent average with standard deviation and statistical significance was assessed with two-tailed *t*-test (***: p=0.001). (C) Fluorescent pictures of AF488-WGA fungal structures and leaves counterstained with calcofluor white [λexcitation (nm): 365; λemission (nm): 420] at 4 dpt. One representative picture of each treatment is shown. Scale bar equals 100 µm.

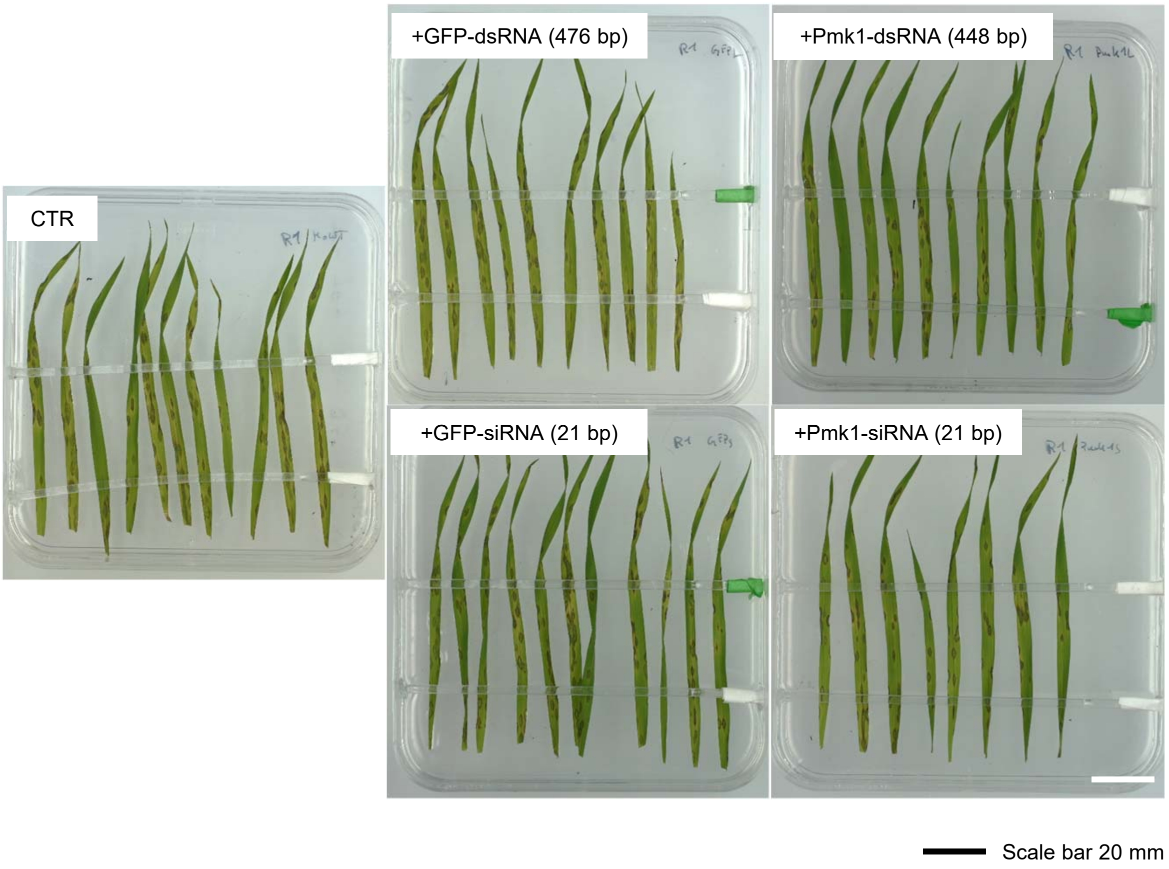

Figure S4. Spray-induced protection of *Brachypodium distachyon* against *Magnaporthe oryzae* infection. Intact three-week-old *Bd* seedlings were sprayed with a solution containing conidia (65×10^3 conidia mL^{-1}) and 0.03 ng/µL of the indicated dsRNA or siRNA. Infection symptoms were determined at 6 dpi. Scale bar = 20 mm.

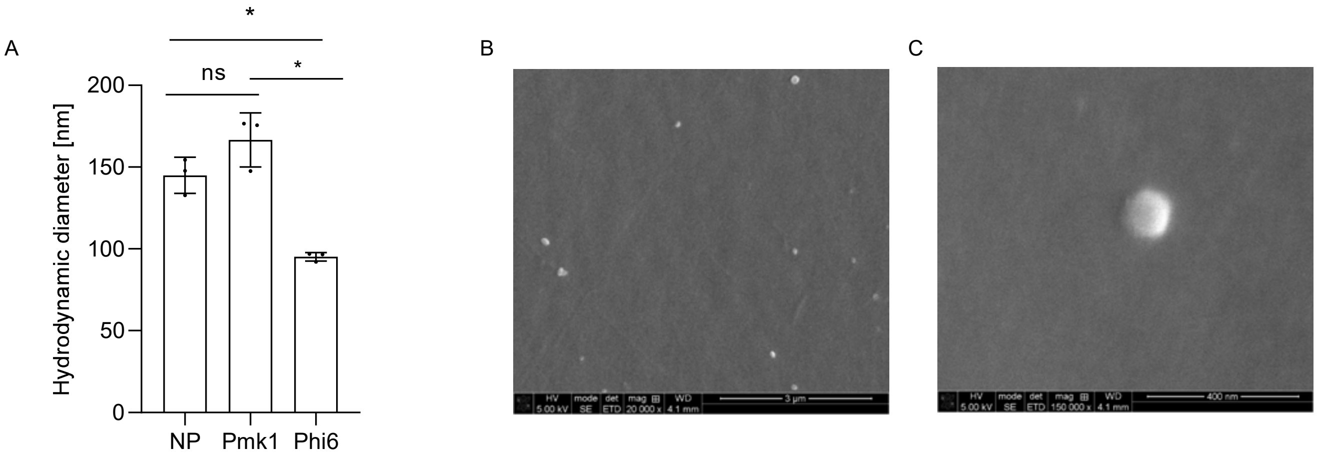

Figure S5. Properties of novel alginate-chitosan nanoparticles (NPs) with dsRNA cargo. (A) Mean hydrodynamic diameter of alginate-chitosan (NPs), Pmk1-dsRNA-alginate-chitosan (Pmk1-NPs) and Phi6-dsRNA-alginate-chitosan nanoparticles (Phi6-NPs) of n = 3 samples determined by dynamic light scattering. Statistical significance according to Welch's ANOVA ($p \leq 0.05$) and Dunnett's post-hoc test (* NPs to Phi6-NPs: $p=0.027$; * Pmk1-NPs to Phi6-NPs: $p=0.033$). (B - C) Scanning electron microscope images of Phi6-dsRNA-alginate-chitosan NPs which were desiccated on polystyrene surface and spattered with a thin layer of tantalum followed by ruthenium. Scale bars are 3 µm and 400 nm for (B) and (C), respectively.
