# Supplementary material for: Exogenous dsRNA triggers sequence-specific RNAi and fungal stress responses to control Magnaporthe oryzae in Brachypodium distachyon
Source: Commun Biol. 2025 Jan 25;8:121. doi: 10.1038/s42003-025-07554-6 (PMC11762700; doi:10.1038/s42003-025-07554-6)
Supplement: Supplementary file 3 — Description of Additional Supplementary File [file 42003_2025_7554_MOESM3_ESM.pdf]

Description of Additional Supplementary Files

File name: Supplementary Data 1

Description: All the data set underlying the graphs.

File name: Supplementary Data 2

Description: DNA sequences for fluorescent labeling.

File name: Supplementary Data 3

Description: DNA sequences of targets for dsRNA production.

File name: Supplementary Data 4

Description: Primer sequences.
